# Distribution of 10-year cardiovascular disease risk levels in Mongolia: results from nation-wide health screening program

**DOI:** 10.3389/fpubh.2025.1412262

**Published:** 2025-06-11

**Authors:** Bayarbold Dangaa, Batzorig Bayartsogt, Ariuntuya Tuvdendorj, Enkhbold Sereejav, Khurelbaatar Nyamdavaa, Damdindorj Boldbaatar, Enkhtur Yadamsuren, Otgonbat Altangerel, Oyuntugs Byambasukh, Narantuya Davaakhuu, Tumur-Ochir Tsedev-Ochir, Mijidsuren Ganbat, Munkhtulga Gantulga, Davaalkham Dambadarjaa, Oyunsuren Enebish

**Affiliations:** 1 Ministry of Health of Mongolia, Ulaanbaatar, Mongolia; 2 Mongolian National University of Medical Sciences, Ulaanbaatar, Mongolia; 3 The Third State Central Hospital, Ulaanbaatar, Mongolia

**Keywords:** screening, cardiovascular disease, public health, mass screen, cardiovascular disease risk

## Abstract

**Introduction:**

The Mongolian government recently initiated the first nationwide health screening program to assess the health status of the entire population using age-group specific health risk assessments related to the major noncommunicable diseases and behavioral risk factors. Based on data collected from nationwide health screening program, we estimated the distribution of 10-year cardiovascular disease (CVD) risks in the general population by using the risk prediction model recently published by the World Health Organization (WHO).

**Methods:**

A representative sample of 35,769 adults aged between 40 and 80 years old participated in nationwide health screening program, which covered all 21 Mongolian provinces and 9 districts in the capital city Ulaanbaatar. Data used in the present study were obtained from the first wave of the screening program, which was held throughout Mongolia between May 2022 and February 2023. To assess the four categories of CVD risk and determine the 10-year risk of a fatal/non-fatal event, we used the WHO CVD risk 2019 laboratory-based cardiovascular disease risk charts. The following key risk factors were used to assess the 10-year CVD risk: gender, age, location, smoking status, body mass index, total cholesterol level, history of diabetes, fasting glucose level, and blood pressure level. The 10-year risk for CVD was divided into four groups: low risk (< 10%), mild risk (10 to 20%), moderate risk (20 to 30%), and high risk (> 30%).

**Results:**

Total of 35,769 adults aged between 40 and 80 years old participated in the nationwide health screening program. The mean age of the participants in the screening program was 54.4 ± 9.3 years. According to the CVD charts published by the WHO, about 48% (*n* = 17,282) of the study population had a low 10-year CVD risk (<10%), 41% (*n* = 14,554) had a mild risk (10–20%), 12% (*n* = 3,502) had a moderate risk (20–30%), and 1% (*n* = 431) had a high risk (>30%).

**Conclusion:**

More than half of the screening program participants were identified as having a 10-year CVD risk greater than 10%. Effective intervention focusing on the male population living in urban areas, and target on major risk factors like hypertension, smoking and diabetes can be strategic approach to reducing the CVD risk in Mongolia.

## 1 Introduction

Every year, about 18 million people die from cardiovascular disease (CVD), representing almost one-third of all global deaths (1). Approximately, 70% of CVD cases and deaths can be attributed to modifiable risk factors such as tobacco use, unhealthy diet, physical inactivity, obesity, elevated blood pressure, abnormal blood lipids and elevated blood glucose (2). Thus, the development of CVD risk in individuals depends largely on their overall risk-factor profile (3).

Mongolia is a landlocked country located between Russia to the north and China to the South. Its total population of 3.4 million is affected by a severe noncommunicable disease (NCD) burden (4).

NCDs account for nearly 80% of the approximately 18,000 deaths that occur annually in Mongolia (5). CVD is the first leading cause of death in Mongolia, accounting for approximately 5,500 to 6,000 deaths each year – representing over one-third of the overall annual 18,000 deaths per year in Mongolia. Men represent 60% of these deaths, while women account for the remaining 40%. Notably, around 92% of CVD-related deaths occur among individuals aged 45 years and older, highlighting its concentration in the older population (5).

Over the past decade, CVD has consistently ranked among the top causes of illness in both outpatient and inpatient health services; CVD has ranked third among the five leading causes of outpatient visits, following respiratory system diseases and digestive system diseases. From 2013 to 2023, CVD has ranked second among all diseases treated in hospitals, just after respiratory diseases.

In terms of incidence, there has been a noticeable upward trend. The number of CVD cases per 10,000 population has increased from 881 in 2013 to 1,060 in 2023, indicating a 20% rise over the last decade.

Similar to other low and middle-income countries (LMIC), the prevalence of behavioral risk factors in the Mongolian population – including tobacco use, excessive alcohol consumption, physical inactivity, unhealthy diet, and excess body weight – has risen (6, 7). As the country undergoes rapid development, lifestyle-related health challenges, especially an increased prevalence of CVD, have emerged. Understanding the specific risk factors for CVD has therefore become essential.

In 2022, the Mongolian government launched a nationwide health screening program to assess the health status of the entire population through age-group-specific health risk assessments related to the major types of NCD and behavioral risk factors (8). The health screening program is funded by the national health insurance scheme, and people throughout the country participated (9). The ultimate goals of this nationwide health screening program are to reduce the burden of disease, to detect disease risk earlier, improve population health, and inform decision makers on the key issues to be considered when planning health policy (10).

The health screening packages designed for individuals aged 40 to 80 years consists of various risk assessment tests. A comprehensive set of health-risk assessments includes a mental health score, tests for tuberculosis, diabetes, cardiovascular risk, metabolic risk, glucose level, Hepatitis B and C, an electrocardiogram, an ultrasound of the abdomen, thyroid and breast, and tests for cervical and esophageal cancers. The nationwide health screening packages are tailored to the abovementioned age groups and consist of risk assessment questionnaires, laboratory tests, and physical examinations. Interviews are performed by trained administrative officers using health-risk assessment questionnaires to collect information on aspects such as demographic status, socioeconomic status, medical history, family history, and behavioral risk factors (smoking status, use of alcohol, physical exercise, and diet). Laboratory tests include blood and urine tests including biochemical and Hepatitis B and C screenings. Physician examinations included 12 measures: height, weight, waist size, body mass index (BMI), blood pressure and heart rate.

The present study focused on CVD risk. Previous research has shown that the distribution of 10-year CVD risk varies significantly between countries. For example, the proportion of people aged 40 to 64 years who are at high risk of CVD ranges from 11% for Latin America to 30% in Central Asia (11). Understanding the distribution of laboratory-based CVD risk in the general population in LMICs, including Mongolia, is essential to its effective management, and for supporting appropriate lifestyle interventions and drug treatment based on patient’s total risk status.

As part of global technical initiative, a CVD risk prediction model that is applicable to LMICs was recently developed and updated (12). An updated CVD risk prediction chart was published for 21 regions, including Central Asia. A laboratory-based chart for this region was also provided, which includes individual-level information on age, gender, smoking status, blood pressure, diabetes mellitus, and total cholesterol value. However, there is a lack of country-specific evidence on proportion of population suffering from CVD at a younger age, and applying the updated CVD risk prediction chart designed for the Asian population. The data from the aforementioned population-based screening program could be used to provide this evidence for Mongolia.

In the present study we used the risk prediction model recently published by the World health organization (WHO) to estimate the distribution of 10-year CVD risk in the general population in Mongolia based on data collected from the population-based screening program.

## 2 Methods

### 2.1 Study design and setting

This study employed a cross-sectional study design using data from the nationwide health screening program to assess the prevalence and the distribution of 10-year CVD risk in Mongolian population. The nationwide health screening program was conducted by the Ministry of Health in Mongolia between 2022 to 2023.The first wave of health screen program included 209,055 adults aged over 18 years. Participants with missing data, outliers in key variables, and those aged below 40 years or above 80 years were excluded, resulting in a final sample of 35,769 adults.

A representative sample of 35,769 adults aged 40 to 80 years old participated in the nationwide health screen program, which included participants from all 21 provinces and 9 districts of the capital city, Ulaanbaatar. A total of 39,237 people aged 40–80 years participated in the screening, out of whom 3,468 were excluded due to incompleteness of questionnaire and tests. As a result, a data analyses were performed for the remaining 35,769 participants with complete screening data. A detailed information of the nationwide health screen program has been described in the previous study (13).

### 2.2 Screening program participants

The nationwide health screening program in Mongolia employed a population-wide approach, inviting all adults aged 18 years and older to participate. Screenings were conducted across all regions and districts, ensuring full geographic coverage. All the Mongolian residents aged 40–80 years were invited and all who wanted to participate in the national screening were enrolled. In other words, a convenient participation method was used. Invitations were disseminated through multiple channels, including the E-Mongolia digital platform, local health centers, and community outreach campaigns. Participation was voluntary but actively promoted by the Ministry of health and local health authorities.

Eligibility for participation required individuals to have paid the national health insurance premium regularly since 2019. All insured individuals received personalized invitations via mobile phone messages, the E-Mongolia platform, or official letters from their family health centers (soum hospitals in rural areas), along with clear instructions on how to take part in the screening and the necessary preparations prior to their health center or hospital visit.

### 2.3 Standardization of data collection

A national screening coordination committee was created in 2021, that was led by the Head of the Department of the Public Health policy of the Ministry of Health. The coordination committee team consisted of cardiovascular professional committee members, national association of cardiovascular diseases and health professionals. The committee developed the methodology and protocol for cardiovascular screening. In order to standardize the screening methods in urban and rural areas, the trainings to standardize data collection method, performance of screening tests were conducted for doctors and healthcare professionals by trained personnel. As a result, total of 2,942 doctors and over 10,000 healthcare professionals were trained in-person and online, respectively. In addition to the standard protocol, and training, all the measurements and tests used in the screening were same in urban and rural areas.

### 2.4 Variables

In our study we included participants who received screening packages for adults between 40 and 80 years old. Mean age and standard deviation (SD) were calculated for both genders. Screening program participants could choose either male or female as their gender identity. The current home address was used to determine the location of the participants. Those living in the capital were classified as urban residents, and those living in the provinces were classified as rural residents. Regarding smoking status, participants were asked to choose between ever-smoker or never-smoker. Those who did not smoke during the last 12 months were instructed to choose the never-smoker option. BMI was calculated by dividing an individual’s weight in kilograms by the square of their heigh in meters. Mean and SD values for BMI were measured for both genders and classified into four groups: underweight (below 18.5 kg/m2), normal weight (18.5 to 24.9), overweight (25 to 29.9), and obese (30 or above). In this study, units of cholesterol were measured in millimoles per liter (mmol/L), and mean and standard deviation of the total cholesterol were calculated for both genders.

The WHO guidelines were used to assess the fasting blood glucose of the participants in mmol/l, and the results were classified into three groups: normoglycemia (≤ 5.6 mmol/L), impaired fasting glucose (5.6–6.9 mmol/L), and diabetes indication (≥7 mmol/L) (14).

Survey participants were asked “Have you even been diagnosed with diabetes?” Two responses were possible: “Yes” which indicates that he/she has been previously diagnosed with diabetes, and “No,” which indicates that he/she has not been previously diagnosed with diabetes.

During the physical examination the participants’ blood pressure was measured and mean and SD values for systolic and diastolic blood pressure were determined in millimeters of mercury (mmHg). Certified electronic sphygmomanometers were used. Measurements were performed three times sequentially on the right arm of the participants in seated position according to protocol (13). Blood pressure was classified into four categories: normal (<120/80 mmHg), elevated (120–129/<80 mmHg), grade 1 hypertension (130–139/80–89 mmHg), and grade 2 hypertension (≥10/90 mmHg).

### 2.5 Data sources/measurement

We calculated the 10-year risk of a fatal/non-fatal event using the cardiovascular disease risk laboratory-based risk charts of the WHO CVD risk 2019 calibrated for use in Central Asia (11). Based on the CVD risk charts, we developed an application to automate and facilitate the risk calculation. The methodogloy to calculating CVD event were explained in Supplementary material. Key risk factors used to assess the 10-year risk for CVD with laboratory-based measures were the following: gender, age, location, smoking status, BMI, total cholesterol level, history of diabetes, fasting glucose level, and blood pressure level. The 10-year risk for CVD was classified into four groups < 10% for low risk, 10 to 20% for mild risk, 20 to 30% for moderate risk, and > 30% for high risk (15).

### 2.6 Statistical analysis

Data analysis was performed using R studio. Descriptive statistics were computed for all variables, including means for continuous variables, frequencies for categorical variables, and standard deviations of the mean values. We conducted stratified analysis by gender, 10-year CVD risk, location and age and then classified the outcomes based on gender, age, location, smoking status, BMI, total cholesterol level, history of diabetes, fasting glucose level, and blood pressure level, with *p*-values. Prevalence values for categorical variables were compared using the χ2 test for proportions and stratified by age group, gender, location, and other potential risk factors. Statistical significance was established at *p* < 0.05.

## 3 Results

Table 1 describes the general characteristics of all adults over 40 years old in the nationwide health screening program throughout Mongolia. A total of 35,769 adults (22,651 female and 13,118 male) with a mean age of 54.4 years participated in nationwide health screening program. Of the 35,769 adults, 20,605 (57.6%) lived in rural areas, and 6,740 (18.8%) were ever-smokers. The mean body mass index was 27.8 ± 4.8, and mean cholesterol level 5.4 ± 1.12 for both genders. Approximately 86% of all adults had normoglycemia fasting blood glucose levels, while 6% had impaired fasting, and 8% were newly diagnosed with diabetes during the medical examination. Exact *p*-values for statistical tests are available in Supplementary Table S1.

**Table 1:** General characteristics of the study participants, expressed as means (95% CI).

Variables	Total (*N* = 35,769)	Female (*N* = 22,651)	Male (*N* = 13,118)	*p*-value
Age, mean (SD)	54.40 (9.29)	54.11 (9.14)	54.90 (9.53)	< 0.001 (1)
Education				< 0.001 (2)
No formal education	1,482 (4.1%)	867 (3.8%)	615 (4.7%)	
Secondary school	22,703 (63.5%)	13,751 (60.7%)	8,952 (68.2%)	
University degree	10,368 (29.0%)	7,175 (31.7%)	3,193 (24.3%)	
Graduate degree	1,216 (3.4%)	858 (3.8%)	358 (2.7%)	
Location				0.481 (2)
Rural	20,605 (57.6%)	13,016 (57.5%)	7,589 (57.9%)	
Urban	15,164 (42.4%)	9,635 (42.5%)	5,529 (42.1%)	
Smoking status				< 0.001 (2)
Never smoker	29,029 (81.2%)	21,633 (95.5%)	7,396 (56.4%)	
Ever smoker	6,740 (18.8%)	1,018 (4.5%)	5,722 (43.6%)	
BMI, Mean (SD)	27.83 (4.83)	27.99 (4.99)	27.56 (4.52)	< 0.001 (1)
BMI				< 0.001 (2)
Underweight	283 (0.8%)	195 (0.9%)	88 (0.7%)	
Normal	10,325 (28.9%)	6,456 (28.5%)	3,869 (29.5%)	
Overweight	14,607 (40.8%)	9,001 (39.7%)	5,606 (42.7%)	
Obese	10,554 (29.5%)	6,999 (30.9%)	3,555 (27.1%)	
Total cholesterol				0.124 (1)
Mean (SD)	5.40 (1.12)	5.40 (1.11)	5.42 (1.13)	
Fasting blood glucose (mmol/l)				< 0.001 (1)
Mean (SD)	5.32 (1.59)	5.21 (1.46)	5.51 (1.78)	
Fasting blood glucose (mmol/l)				< 0.0001 (2)
Normoglycemia	30,757 (86.0%)	19,913 (87.9%)	10,844 (82.7%)	
Impaired fasting glucose	2,137 (6.0%)	1,139 (5.0%)	998 (7.6%)	
Newly diagnosed diabetes	2,875 (8.0%)	1,599 (7.1%)	1,276 (9.7%)	
Blood pressure (mmHg)				
Diastolic, mean (SD)	80.60 (11.52)	79.83 (11.60)	81.93 (11.27)	< 0.001 (1)
Systolic, mean (SD)	124.93 (17.16)	123.90 (17.36)	126.74 (16.66)	< 0.001 (1)
Blood pressure				< 0.001 (2)
Normal	9,050 (25.3%)	6,419 (28.3%)	2,631 (20.1%)	
Elevated	1719 (4.8%)	1,131 (5.0%)	588 (4.5%)	
Grade 1	18,565 (51.9%)	11,302 (49.9%)	7,263 (55.4%)	
Grade 2	6,435 (18.0%)	3,799 (16.8%)	2,636 (20.1%)	

1. T test.

2. Pearson’s Chi-squared test.

Female and male participants differed significantly with respect to education, smoking status, body mass index, diabetes, fasting glucose level and blood pressure. For example, male participants smoked 10 times more frequently higher than females, had a higher body mass index and higher blood pressure level.

Table 2 shows the distribution of 10-year CVD risks classified as <10, 10–20%, 20–30% and >30%. More than half of the participants in the screening program were identified as having >10% risk. The prevalence of 10-year CVD risk increased with 10-year age groups. The mean age of the group with less than 10% risk was 52.5 years, and it was increased to 58.2 years when having more than 30% risk. The proportion of individuals with an estimated risk greater than 20% varied by location, from 6% for rural residents to 14.9% for urban residents. Exact *p*-values for statistical tests are available in Supplementary Table S2.

**Table 2:** Distribution of 10-year CVD risk *n* (%) or mean (95%CI).

Variables	<10% (*N* = 17,282)	10–20% (*N* = 14,554)	20–30% (*N* = 3,502)	>30% (*N* = 431)	*p*-value
Age, mean (SD)	52.52 (8.78)	55.48 (9.27)	58.71 (9.51)	58.25 (9.23)	< 0.001 (1)
Education					< 0.001 (1)
No formal education	783 (52.8%)	573 (38.7%)	110 (7.4%)	16 (1.1%)	
Secondary school	11,079 (48.8%)	9,203 (40.5%)	2,161 (9.5%)	260 (1.1%)	
University degree	4,851 (46.8%)	4,311 (41.6%)	1,067 (10.3%)	139 (1.3%)	
Graduate degree	569 (46.8%)	467 (38.4%)	164 (13.5%)	16 (1.3%)	
Location					< 0.001 (2)
Rural	11,410 (55.4%)	7,794 (37.8%)	1,245 (6.0%)	156 (0.8%)	
Urban	5,872 (38.7%)	6,760 (44.6%)	2,257 (14.9%)	275 (1.8%)	
Smoking status					< 0.001 (2)
Never-smoker	14,155 (48.8%)	11,801 (40.7%)	2,746 (9.5%)	327 (1.1%)	
Ever-smoker	3,127 (46.4%)	2,753 (40.8%)	756 (11.2%)	104 (1.5%)	
BMI, mean (SD)	27.20 (4.58)	28.21 (4.88)	29.17 (5.25)	29.63 (5.09)	< 0.001 (1)
BMI					< 0.001 (2)
Underweight	166 (58.7%)	96 (33.9%)	18 (6.4%)	3 (1.1%)	
Normal	5,735 (55.5%)	3,799 (36.8%)	722 (7.0%)	69 (0.7%)	
Overweight	7,147 (48.9%)	5,936 (40.6%)	1,358 (9.3%)	166 (1.1%)	
Obese	4,234 (40.1%)	4,723 (44.8%)	1,404 (13.3%)	193 (1.8%)	
Total cholesterol					< 0.001 (1)
Mean (SD)	5.29 (1.05)	5.45 (1.13)	5.71 (1.22)	5.95 (1.59)	
Fasting blood glucose (mmol/l)					< 0.001 (1)
Mean (SD)	5.16 (1.25)	5.38 (1.64)	5.78 (2.33)	6.19 (2.90)	
Fasting blood glucose (mmol/l)					< 0.001 (2)
Low fasting glucose	804 (51.2%)	599 (38.2%)	151 (9.6%)	16 (1.0%)	
Normal fasting glucose	14,874 (50.1%)	11,865 (40.0%)	2,627 (8.9%)	300 (1.0%)	
Increased fasting glucose	917 (39.6%)	1,105 (47.8%)	251 (10.9%)	40 (1.7%)	
Diagnosed diabetes	687 (30.9%)	985 (44.4%)	473 (21.3%)	75 (3.4%)	
Diabetes					< 0.001 (2)
No	16,832 (49.1%)	13,907 (40.6%)	3,183 (9.3%)	368 (1.1%)	
Yes	450 (30.4%)	647 (43.7%)	319 (21.6%)	63 (4.3%)	
Blood pressure (mmHg)					< 0.001 (1)
Diastolic, mean (SD)	78.99 (11.23)	81.34 (11.34)	85.00 (11.80)	84.44 (13.84)	
Systolic, mean (SD)	122.13 (16.32)	126.31 (17.03)	131.97 (18.11)	132.91 (20.87)	
Blood pressure					< 0.001 (2)
Normal	5,506 (60.8%)	3,090 (34.1%)	366 (4.0%)	88 (1.0%)	
Elevated	927 (53.9%)	660 (38.4%)	120 (7.0%)	12 (0.7%)	
Grade 1	8,526 (45.9%)	7,895 (42.5%)	1960 (10.6%)	184 (1.0%)	
Grade 2	2,323 (36.1%)	2,909 (45.2%)	1,056 (16.4%)	147 (2.3%)	

1. One way anova test.

2. Pearson’s Chi-squared test.

In the group with less than 10% risk, the mean total cholesterol was level was about 5.3, while it increased to 6 for the group with more 30% risk. In the group with an estimated CVD risk greater than 20%, about 4% of the individuals had normal blood pressure and 16.4% had grade 2 hypertension.

**Figure 1:**
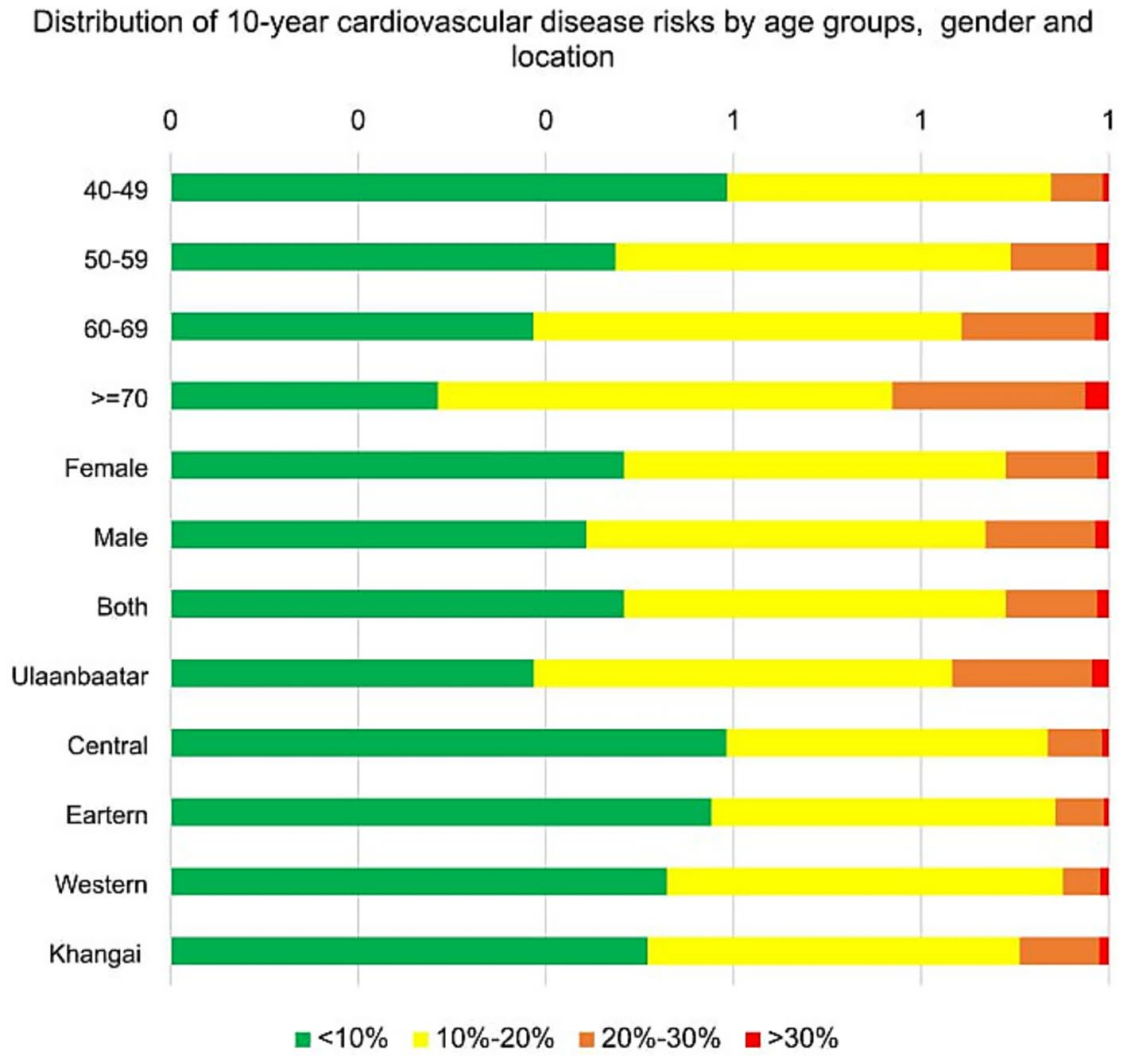
Distribution of 10-year cardiovascular disease risks by age groups, gender and location.

Figure 1 illustrates the estimated distribution of 10-year CVD risk in the sample population aged between 40 and 80 years old, stratified by 10-year age groups, gender and location.

Overall, the proportion of participants with a higher CVD risk increased in every 10-year age group (from younger to older). Almost half (48%) of the females had low CVD risk (<10%) while 41% had 10–20% risk, 10% had 20–30% risk, and 1% had >30% risk. Of the males, 44% had low CVD risk (<10%), 43% had 10–20% risk, 12% had 20–30% risk, and 1% had >30% risk.

The highest proportion of participants with increased CVD risk was found in male residents of Ulaanbaatar city. In females with more than 20% risk, about 5% were identified in the youngest age group, while this increased to 23% in oldest age group.

**Figure 2:**
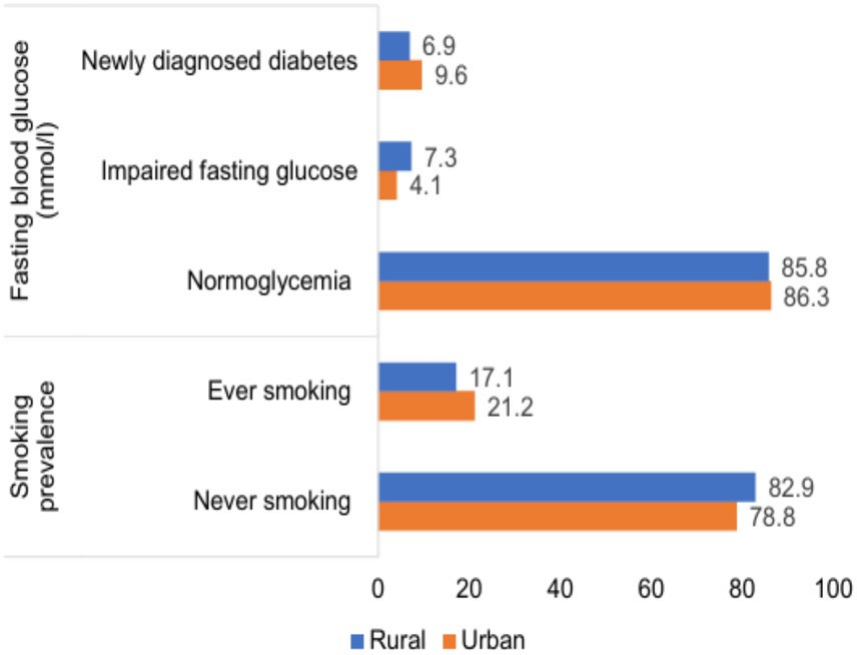
Percentage of major risk factors for 10-year CVD risks.

Figure 2 shows the major risk factors contributing to the increased 10-year CVD risk in urban populations. These risk factors were prevalence of smoking and newly diagnosed diabetes during the screening program.

## 4 Discussion

This study was based on data from the large representative sample of 35,769 participants aged between 40 and 80 years old who participated in the first wave of the nationwide health screening program in Mongolia. This is the first study in Mongolia to use the laboratory-based prediction model for 10-year CVD risk that was published by the WHO CVD chart. This model was designed in 2019, developed for Central Asian countries, including Mongolia.

We found that almost half of the sample population (aged between 40 and 80 years) had a CVD risk of more than 10%, and that 11% had a risk of more than 20%. For females, the proportion of participants in the oldest age group (70–80 years) that were identified as having a > 20% CVD risk was 4.6 times higher than in the youngest age group (40–49 years). For males, this proportion was 2.3 times higher. We found large differences in the proportion of the population with >10% CVD risk between regions in Mongolia, with lowest proportion in Central region and the highest in Khangai region. The proportion of participants with >20% CVD risk was highest in the capital city Ulaanbaatar, where almost half of the total population of Mongolia resides. These differences between various groups could be partially explained by differences in the prevalence of potential risk factors, regional environments, sedentary lifestyles and other socioeconomic factors in Mongolia.

Multiple individual-level risk factors – including smoking, hypertension, and diabetes – increase the risk for developing CVD (12). A previous study found that hypertension, smoking, and diabetes are the main risk factors for CVD in Asia (16). Differences in smoking prevalence are substantial in Mongolia, where about half of the men smoke compared to only 5% of the women (17). Tobacco Control Law, originally approved in 1993, has been revised four times over the past two decades to strengthen its regulations. This law includes a comprehensive range of tobacco control measures, such as smoke-free public places, restriction on the sale, mass media campaigns, ban on tobacco advertising and tobacco taxes.

Population living in urban face risk factors associated with unhealthy lifestyles, high level of smoking and diabetes, while rural struggle with healthcare access and traditional dietary pattern. The government of Mongolia approved policy on “Regional development concept on Mongolia” on declaring 20,224 as the Year to support regional development in Mongolia. The aim of the policy was to reduce regional social and geographic disparities between the urban and rural areas, fostering economic development and improving the quality of life across the country.

According to the health screening program, the prevalence of smoking was higher in urban as compare to rural in Mongolia, which was 21.2% in urban versus 17.1% for rural among the screening program participants aged between 40 and 80. This finding was comparable with the recent national survey of the major risk factors for noncommunicable diseases (STEP) in Mongolia. According to the STEP survey, the prevalence of smoking in urban was 25%, while it was 22.5% in rural population aged 15 and 64 years old (18).

The estimated 10-year CVD risk varied substantially across the countries, being lowest in South Korea, Spain, and Denmark where about 5–10 percent of men and women had >10% CVD risk. On the other hand, China reports higher level of 10-year CVD where 33% of men and 28% of women had > 10% CVD risk (19). The high CVD risk in Mongolia, when compared to other countries, can be attributed to the combination of environmental factors, life style factors and socioeconomic condition.

Our results are consistent with findings from other countries in the region, although the overall burden appears to be higher in Mongolia. For instance, a population-based study in China using the 2019 CVD disease risk chart for east Asia found that 16.6% of adults had a high risk (defined as a 10-year CVD risk is greater than 20%), with regional variation ranging from 2.8 to 34.2% across the countries (20). Notably, the highest CVD risk was observed in northeast China, a region that share a border and similar climatic and lifestyle characteristics with Mongolia. This geographic similarity may partially explain the elevated CVD risk levels observed in our study.

Accurate estimates of CVD risk in the general population are crucial for developing and implementing preventive strategies at the national and regional levels and in primary care. Such estimates would enable practitioners to identify people at high risk of CVD who would benefit the most from preventive interventions (21). Various risk prediction models for CVD have been developed and have led to interventions with wide range of CVD outcomes in the general populations (22).

A previous review summarized 363 prediction models, most of which (46%) originated in Europe or North America (22). The most commonly used risk prediction models for CVD are the Framingham risk score for Americans and the Systematic Coronary Risk Evaluation (SCORE) for Europeans. However, these risk prediction models were largely validated in populations from developed countries (11). In contrast, our model is based on the representative sample of healthy people from all regions of Mongolia who participated in the population-based screening program using hospital-based data. The large size of the study sample (about 33,000 adults) has enabled us to make more precise estimations of the prevalence of 10-year CVD risk in Mongolia. Moreover, in our analysis we used the laboratory-based risk prediction model (recently published by the WHO), which was specifically designed for the Asian population. Thus, our estimations can potentially be used in similar countries that lack data on the 10-year risk for CVD.

Our study utilized a large, nationally representative sample of over 35,000 adults, encompassing all geographical regions of Mongolia. This represents the most comprehensive assessment of cardiovascular disease (CVD) risk conducted in the country to date. We applied the most recent laboratory-based CVD risk prediction model developed by the World Health Organization (WHO) for the Central Asia region, enabling internationally comparable estimates. The data were derived from a nationwide, government-led screening program, which included a large and diverse population from both urban and rural settings, ensuring broad representativeness of Mongolia’s adult population. Although the program did not use random sampling, its extensive scale and inclusive design provided wide coverage across demographic, geographic, and socioeconomic groups.

The main limitation of our study is that inclusion in the health screening program required that participants were insured by the national health insurance program during the previous 3 years. Although, the national health insurance program in Mongolia has almost universal coverage (89% in 2018), the exclusion of non-insured people from the study population could affect the accuracy of our estimations (23). Our study relied on baseline data from a single round of the nationwide screening program, which limits the ability to assess temporal trends or changes in CVD risk over time. Additionally, we excluded adults under 40 and over 80 years of age in accordance with the age range specified in the WHO CVD risk prediction model. This exclusion may result in an underestimation of the burden among younger adults, particularly those with early-onset risk factors. Moreover, the risk model used does not account for important variables such as family history of CVD, socioeconomic status, or psychosocial factors, which may influence individual and regional risk estimates.

A previous study in Mongolia reported that several criteria for offering efficient interventions for CVD, such as providing drug therapy and counseling services for persons at greater than 20% risk of a fatal CVD event in the next 10 years, were not achieved (24).

In Mongolia there is a substantial need to mitigate the 10-year risk of developing CVD, which could be done by implementing personalized preventive measures in primary and secondary care. These measures should focus on the male population and on the urban area, and on risk factors for CVD such as hypertension, smoking, and diabetes. Further research is required to identify the most effective preventive measures for the target population, but these measures will likely involve promoting healthy lifestyles, using evidence-based treatments, and providing access to essential medicines and technology.

## 5 Conclusion

More than half of the screening program participants were identified as having a 10-year CVD risk greater than 10%. Effective intervention focusing on the male population living in urban areas, and target on major risk factors like hypertension, smoking and diabetes can be strategic approach to reducing the CVD risk in Mongolia.

## Data Availability

The raw data supporting the conclusions of this article will be made available by the authors, without undue reservation.
