# Correction: Distribution of 10-year cardiovascular disease risk levels in Mongolia: results from nation-wide health screening program

**DOI:** 10.3389/fpubh.2025.1747397

**Published:** 2025-11-26

**Authors:** Bayarbold Dangaa, Batzorig Bayartsogt, Ariuntuya Tuvdendorj, Enkhbold Sereejav, Khurelbaatar Nyamdavaa, Damdindorj Boldbaatar, Enkhtur Yadamsuren, Otgonbat Altangerel, Oyuntugs Byambasukh, Narantuya Davaakhuu, Tumur-Ochir Tsedev-Ochir, Mijidsuren Ganbat, Munkhtulga Gantulga, Davaalkham Dambadarjaa, Oyunsuren Enebish

**Affiliations:** 1 Ministry of Health of Mongolia, Ulaanbaatar, Mongolia; 2 Mongolian National University of Medical Sciences, Ulaanbaatar, Mongolia; 3 The Third State Central Hospital, Ulaanbaatar, Mongolia

**Keywords:** screening, cardiovascular disease, public health, mass screen, cardiovascular disease risk

Affiliation 2 “Mongolian National University of Medical Sciences, Ulaanbaatar, Mongolia” was erroneously omitted for author Bayarbold Dangaa. This affiliation has now been added for author Bayarbold Dangaa.

The original version of this article has been updated.

