# Supplementary material for: Distribution of 10-year cardiovascular disease risk levels in Mongolia: results from nation-wide health screening program
Source: Front Public Health. 2025 Jun 11;13:1412262. doi: 10.3389/fpubh.2025.1412262 (PMC12187829; doi:10.3389/fpubh.2025.1412262)
Supplement: Supplementary file 1 [file Table_1.DOCX]

# **DISTRIBUTION OF 10-YEAR CARDIOVASCULAR DISEASE RISK LEVELS IN MONGOLIA: RESULTS FROM NATION-WIDE HEALTH SCREENING PROGRAM**

Total of 8 steps were followed when using the WHO CVD risk charts during the screening program.

1. Select the chart as relevant for people with or without diabetes.
2. Select the table for men and women, as appropriate
3. Select the smoker or non-smoker column
4. Select the age-group
5. Within the selected box find the cell where the person’s systolic blood pressure and total blood cholestrol intersect.
6. The color of the cell indicate 10-year CVD risk event. Colour coding is based on the grouping.

| Low | ≤ 10% |
| --- | --- |
| Moderate | 10-20% |
| High | 20-30% |
| Very high | ≥30% |

1. Record CVD risk percentage in person’s chart
2. Counsel, treat and refer according to risk level.

Table S1 General characteristics of the study participants, expressed as means (95% CI)

| Variables | Total (N=35,769) | Female (N=22,651) | Male (N=13,118) | p value |  |
| --- | --- | --- | --- | --- | --- |
| Age, mean (SD) | 54.40 (9.29) | 54.11 (9.14) | 54.90 (9.53) | 1.336e-14† |  |
| Education |  |  |  | 2.120e-58‡ |  |
| No formal education | 1482 (4.1%) | 867 (3.8%) | 615 (4.7%) |  |  |
| Secondary school | 22703 (63.5%) | 13751 (60.7%) | 8952 (68.2%) |  |  |
| University degree | 10368 (29.0%) | 7175 (31.7%) | 3193 (24.3%) |  |  |
| Graduate degree | 1216 (3.4%) | 858 (3.8%) | 358 (2.7%) |  |  |
| Location |  |  |  | 0.481 |  |
| Rural | 20605 (57.6%) | 13016 (57.5%) | 7589 (57.9%) |  |  |
| Urban | 15164 (42.4%) | 9635 (42.5%) | 5529 (42.1%) |  |  |
| Smoking status |  |  |  | 2.2e-16‡ |  |
| Never smoker | 29029 (81.2%) | 21633 (95.5%) | 7396 (56.4%) |  |  |
| Ever smoker | 6740 (18.8%) | 1018 (4.5%) | 5722 (43.6%) |  |  |
| BMI, Mean (SD) | 27.83 (4.83) | 27.99 (4.99) | 27.56 (4.52) | 2.159e-16† |  |
| BMI |  |  |  | 3.854e-14‡ |  |
| Underweight | 283 (0.8%) | 195 (0.9%) | 88 (0.7%) |  |  |
| Normal | 10325 (28.9%) | 6456 (28.5%) | 3869 (29.5%) |  |  |
| Overweight | 14607 (40.8%) | 9001 (39.7%) | 5606 (42.7%) |  |  |
| Obese | 10554 (29.5%) | 6999 (30.9%) | 3555 (27.1%) |  |  |
| Total cholesterol |  |  |  | 0.124† |  |
| Mean (SD) | 5.40 (1.12) | 5.40 (1.11) | 5.42 (1.13) |  |  |
| Fasting blood glucose (mmol/l) | | |  | 5.233e-68† |  |
| Mean (SD) | 5.32 (1.59) | 5.21 (1.46) | 5.51 (1.78) |  |  |
| Fasting blood glucose (mmol/l) | | |  | 2.2e-16† |  |
|  | 30757 (86.0%) | 19913 (87.9%) | 10844 (82.7%) |  |  |
| Impaired fasting glucose | 2137 (6.0%) | 1139 (5.0%) | 998 (7.6%) |  |  |
| Newly diagnosed diabetes | 2875 (8.0%) | 1599 (7.1%) | 1276 (9.7%) |  |  |
| Blood pressure (mmHg) |  |  |  |  |  |
| Diastolic, mean (SD) | 80.60 (11.52) | 79.83 (11.60) | 81.93 (11.27) | 1.761e-62† |  |
| Systolic, mean (SD) | 124.93 (17.16) | 123.90 (17.36) | 126.74 (16.66) | 3.460e-50† |  |
| Blood pressure |  |  |  | 6.402e-71‡ |  |
| Normal | 9050 (25.3%) | 6419 (28.3%) | 2631 (20.1%) |  |  |
| Elevated | 1719 (4.8%) | 1131 (5.0%) | 588 (4.5%) |  |  |
| Grade 1 | 18565 (51.9%) | 11302 (49.9%) | 7263 (55.4%) |  |  |
| Grade 2 | 6435 (18.0%) | 3799 (16.8%) | 2636 (20.1%) |  |  |

1. †T test
2. ‡ Pearson’s Chi-squared test

Table S2 Distribution of 10-year CVD risk n (%) or mean (95%CI)

| **Variables** | **<10% (N=17,282)** | **10%-20% (N=14,554)** | **20%-30% (N=3,502)** | **>30% (N=431)** | **p value** |
| --- | --- | --- | --- | --- | --- |
| Age, mean (SD) | 52.52 (8.78) | 55.48 (9.27) | 58.71 (9.51) | 58.25 (9.23) | 2e-16† |
| Education |  |  |  |  | 8.44e-08‡ |
| No formal education | 783 (52.8%) | 573 (38.7%) | 110 (7.4%) | 16 (1.1%) |  |
| Secondary school | 11079 (48.8%) | 9203 (40.5%) | 2161 (9.5%) | 260 (1.1%) |  |
| University degree | 4851 (46.8%) | 4311 (41.6%) | 1067 (10.3%) | 139 (1.3%) |  |
| Graduate degree | 569 (46.8%) | 467 (38.4%) | 164 (13.5%) | 16 (1.3%) |  |
| Location |  |  |  |  | 2.10e-29‡ |
| Rural | 11410 (55.4%) | 7794 (37.8%) | 1245 (6.0%) | 156 (0.8%) |  |
| Urban | 5872 (38.7%) | 6760 (44.6%) | 2257 (14.9%) | 275 (1.8%) |  |
| Smoking status |  |  |  |  | 6.60e-07‡ |
| Never-smoker | 14155 (48.8%) | 11801 (40.7%) | 2746 (9.5%) | 327 (1.1%) |  |
| Ever-smoker | 3127 (46.4%) | 2753 (40.8%) | 756 (11.2%) | 104 (1.5%) |  |
| BMI, mean (SD) | 27.20 (4.58) | 28.21 (4.88) | 29.17 (5.25) | 29.63 (5.09) | 2.10e-29† |
| BMI |  |  |  |  | 2.40e-13‡ |
| Underweight | 166 (58.7%) | 96 (33.9%) | 18 (6.4%) | 3 (1.1%) |  |
| Normal | 5735 (55.5%) | 3799 (36.8%) | 722 (7.0%) | 69 (0.7%) |  |
| Overweight | 7147 (48.9%) | 5936 (40.6%) | 1358 (9.3%) | 166 (1.1%) |  |
| Obese | 4234 (40.1%) | 4723 (44.8%) | 1404 (13.3%) | 193 (1.8%) |  |
| Total cholesterol |  |  |  |  | 2.10e-26† |
| Mean (SD) | 5.29 (1.05) | 5.45 (1.13) | 5.71 (1.22) | 5.95 (1.59) |  |
| Fasting blood glucose (mmol/l) |  |  |  |  | 2.10e-26† |
| Mean (SD) | 5.16 (1.25) | 5.38 (1.64) | 5.78 (2.33) | 6.19 (2.90) |  |
| Fasting blood glucose (mmol/l) |  |  |  |  | 9.00e-13‡ |
| Low fasting glucose | 804 (51.2%) | 599 (38.2%) | 151 (9.6%) | 16 (1.0%) |  |
| Normal fasting glucose | 14874 (50.1%) | 11865 (40.0%) | 2627 (8.9%) | 300 (1.0%) |  |
| Increased fasting glucose | 917 (39.6%) | 1105 (47.8%) | 251 (10.9%) | 40 (1.7%) |  |
| Diagnosed diabetes | 687 (30.9%) | 985 (44.4%) | 473 (21.3%) | 75 (3.4%) |  |
| Diabetes |  |  |  |  | 7.16e-96‡ |
| No | 16832 (49.1%) | 13907 (40.6%) | 3183 (9.3%) | 368 (1.1%) |  |
| Yes | 450 (30.4%) | 647 (43.7%) | 319 (21.6%) | 63 (4.3%) |  |
| Blood pressure (mmHg) |  |  |  |  |  |
| Diastolic, mean (SD) | 78.99 (11.23) | 81.34 (11.34) | 85.00 (11.80) | 84.44 (13.84) | 2.10e-26 |
| Systolic, mean (SD) | 122.13 (16.32) | 126.31 (17.03) | 131.97 (18.11) | 132.91 (20.87) | 2.10e-26 |
| Blood pressure |  |  |  |  | 1.58e-28‡ |
| Normal | 5506 (60.8%) | 3090 (34.1%) | 366 (4.0%) | 88 (1.0%) |  |
| Elevated | 927 (53.9%) | 660 (38.4%) | 120 (7.0%) | 12 (0.7%) |  |
| Grade 1 | 8526 (45.9%) | 7895 (42.5%) | 1960 (10.6%) | 184 (1.0%) |  |
| Grade 2 | 2323 (36.1%) | 2909 (45.2%) | 1056 (16.4%) | 147 (2.3%) |  |

1. †one way anova test
2. ‡ Pearson’s Chi-squared test
